# Intensive Care Management and Outcomes of Neuroleptic Malignant Syndrome: A Single-Center Retrospective Study

**DOI:** 10.3390/medicina62020378

**Published:** 2026-02-14

**Authors:** Fatma Özdemir, Dicle Birtane, Nurdan Yılmaz, Zafer Çukurova

**Affiliations:** 1 Department of Anesthesiology and Reanimation, Bakırköy Dr. Sadi Konuk Training and Research Hospital, Istanbul 34147, Türkiye; dicle1tane@gmail.com (D.B.); zcukurova@gmail.com (Z.Ç.); 2 Department of Anesthesiology and Reanimation, Medipol Mega University Hospital, Istanbul 34214, Türkiye; nurdanclb@gmail.com

**Keywords:** neuroleptic malignant syndrome, intensive care, mechanical ventilation, acute kidney injury, critical illness

## Abstract

*Background and Objectives:* Neuroleptic malignant syndrome (NMS) is a rare but high-mortality clinical condition. The aim of this study was to describe the demographic characteristics, clinical and laboratory findings, management strategies, and clinical outcomes of adult patients with NMS treated in an adult intensive care unit. *Materials and Methods:* This retrospective descriptive study included adult patients admitted to a tertiary care ICU with a diagnosis of NMS. Data were obtained from medical records and included demographic characteristics, suspected NMS-related drug exposures, clinical and laboratory findings at ICU admission, disease severity indicators, treatments administered, and clinical outcomes. Continuous variables were expressed as median and interquartile range (IQR), and categorical variables as number and percentage. *Results:* A total of 42 patients were included. The median age was 48 years (IQR: 24.1–75.5), and 61.9% were male. Atypical antipsychotics were the most frequently implicated agents (69.0%). Altered mental status was observed in 92.9% of patients, tachycardia in 61.9%, and hyperthermia in 47.6%. Creatine kinase levels >1000 U/L were present in 57.1%, and leukocytosis in 71.4%. Mechanical ventilation was required in 59.5% of patients. Acute kidney injury developed in 31%, and continuous renal replacement therapy (CRRT) was initiated in 21.4%. Bromocriptine was administered in 54.8% of cases, dantrolene in 4.8%, and amantadine in 11.9%. The ICU mortality rate was 9.5%. *Conclusions:* Patients with neuroleptic malignant syndrome often present with a severe clinical course requiring advanced organ support in the intensive care unit. However, with early diagnosis and appropriate intensive care management, mortality can be maintained at acceptable levels.

## 1. Introduction

NMS is a rare but potentially life-threatening neurological syndrome that may develop following the use of antidopaminergic drugs, the abrupt discontinuation of dopaminergic medications, or changes in treatment regimens. The incidence of NMS among patients using antidopaminergic drugs has been reported to range between 0.01% and 3.2%, and mortality rates have been reported to reach 10–20%. NMS may lead to severe critical illness and death. In the literature, the mortality rate has been reported as 7.6% in NMS cases associated with typical antipsychotics and 3.3% in cases associated with atypical antipsychotics [1]. NMS has been reported to occur more frequently in males and young adults; however, age and sex are not considered independent risk factors [2,3,4].

The main agents implicated in the development of NMS include typical antipsychotics (e.g., haloperidol, fluphenazine, chlorpromazine, thioridazine), atypical antipsychotics (e.g., clozapine, risperidone, olanzapine, aripiprazole), and antiemetics that block dopamine receptors (e.g., metoclopramide, domperidone, droperidol, and prochlorperazine) [3,4,5,6,7,8]. In addition, the abrupt discontinuation, dose reduction, or modification of dopamine agonists used in the treatment of Parkinson’s disease may also lead to the development of NMS [9,10].

The diagnostic criteria for NMS have still not been clearly defined. In a consensus in 2011, an attempt was made to define diagnostic criteria; history of dopamine antagonist exposure or dopamine agonist withdrawal, hyperthermia, muscle rigidity, altered mental status, elevation of creatine kinase levels to more than four times the upper limit of normal, sympathetic nervous system lability and exclusion of other possible causes were proposed as basic criteria [11]. However, these criteria have not been validated in prospective studies, and their use in clinical practice is limited.

According to the DSM-5, the diagnosis of NMS is based on the presence of more than one core clinical feature (hyperthermia, muscle rigidity, altered level of consciousness, and autonomic instability) in association with exposure to dopamine antagonists or withdrawal of dopaminergic medications. Supportive laboratory findings include elevated creatine kinase (CK) levels, leukocytosis, metabolic acidosis, and decreased serum iron levels; however, the presence of these findings is not mandatory for diagnosis. The DSM-5 specifically emphasizes that classic features such as hyperthermia or muscle rigidity may not be present in every case and that the absence of these findings does not exclude the diagnosis of NMS. Before establishing the diagnosis, differential diagnoses such as central nervous system infections, serotonin syndrome, malignant catatonia, malignant hyperthermia, and heat stroke should be excluded [12].

The most commonly observed clinical features include altered mental status, hyperthermia, muscle rigidity, and autonomic dysfunction. Altered mental status is often the initial symptom and may progress from stupor to coma [13]. Muscle rigidity may range in severity from mild stiffness to “lead-pipe” rigidity [14]. Hyperthermia is usually above 38 °C and has been reported to exceed 40 °C in some cases. Autonomic dysfunction may manifest as tachycardia, hypertension or hypotension, diaphoresis, dysphagia, tachypnea, and cardiac arrhythmias [15].

The cornerstone of NMS treatment consists of immediate discontinuation of the offending agent and intensive supportive care. In refractory cases, pharmacological agents such as bromocriptine, dantrolene, and amantadine, as well as electroconvulsive therapy (ECT), may be administered. In cases of NMS resulting from the withdrawal of dopaminergic medications, levodopa therapy may be lifesaving. Early recognition and management of complications are critical for prognosis [16,17,18,19,20].

In severe cases of NMS, respiratory failure, acute kidney injury secondary to rhabdomyolysis, and multiple organ dysfunction may develop; therefore, advanced intensive care interventions such as mechanical ventilation and continuous renal replacement therapy (CRRT) may be required. In this study, the clinical management and outcomes of 42 patients diagnosed with Neuroleptic Malignant Syndrome who were treated in our ICU were retrospectively evaluated.

## 2. Materials and Methods

### 2.1. Study Design and Setting

This study was designed as a retrospective observational cohort study. The study was conducted in the adult intensive care unit of Bakırköy Dr. Sadi Konuk Training and Research Hospital, located in Istanbul, Türkiye. This tertiary care center provides advanced healthcare services and consists of a closed 28-bed intensive care unit, admitting approximately 1440 medical, surgical, and trauma patients annually. The nurse-to-patient ratio is 1:2, and advanced life support therapies such as mechanical ventilation, CRRT plasma exchange therapy, and extracorporeal membrane oxygenation (ECMO) are available 24 h a day, 7 days a week.

### 2.2. Inclusion and Exclusion Criteria

Adult patients (≥18 years) admitted to the intensive care unit with a diagnosis of Neuroleptic Malignant Syndrome and with available clinical and laboratory data were included. Patients with incomplete or insufficient medical records were excluded.

### 2.3. Data Collection

Medical records of patients admitted to the intensive care unit with a diagnosis of Neuroleptic Malignant Syndrome between 1 January 2014 and 15 September 2025 were obtained using Structured Query Language (SQL) queries through the EMRall-QlinICU ImdSoft Metavision Clinical Decision Support System and were retrospectively reviewed.

Collected data included demographic characteristics, Glasgow Coma Scale (GCS) score at ICU admission, heart rate, body temperature, presence of muscle rigidity, oxygen saturation, arterial blood pressure values, white blood cell count, creatine kinase level, length of ICU stay, requirement for and duration of mechanical ventilation, need for CRRT, ICU mortality, past medical history, suspected NMS-related drug exposures, and medical treatments administered in the ICU.

Before admission to the intensive care unit with a diagnosis of NMS, all patients were evaluated by psychiatry and neurology specialists, and differential diagnoses and cranial imaging were completed prior to ICU admission. This approach reduced the risk of “overdiagnosis” in the diagnostic process. The diagnosis of Neuroleptic Malignant Syndrome was established based on DSM-5 diagnostic criteria. Individual demographic, clinical, and laboratory data for all included patients are provided in Supplementary Table S1.

### 2.4. Outcome Measures

The primary outcome of the study was ICU mortality. Secondary outcomes were defined as length of ICU stay, requirement for mechanical ventilation, and need for renal replacement therapy.

### 2.5. Statistical Analysis

Descriptive statistics were used in this study. The distribution of continuous variables was assessed using the Shapiro–Wilk test. Variables with normal distribution were presented as mean ± standard deviation, while variables with non-normal distribution were presented as median (interquartile range, IQR). Categorical variables were expressed as number and percentage [n (%)].

Clinical and laboratory findings were defined as binary variables according to predefined threshold values: hyperthermia (>38 °C) tachycardia (≥100 beats/min), hypotension (mean arterial pressure ≤65 mmHg), hypertension (mean arterial pressure ≥110 mmHg), altered mental status (Glasgow Coma Scale <15), elevated creatine kinase (>1000 U/L), and leukocytosis (white blood cell count >10.0 × 10^9^/L).

All statistical analyses were performed using IBM SPSS Statistics for Windows, Version 26.0 (IBM Corp., Armonk, NY, USA).

## 3. Results

A total of 42 patients diagnosed with NMS who were followed in the adult intensive care unit were included in the study. The median age of the patients was 48.0 years (IQR: 24.1–75.5), and 26 patients (61.9%) were male.

When medication exposures associated with the development of NMS were evaluated, atypical antipsychotic use was identified in 29 patients (69.0%), combined use of typical and atypical antipsychotics in 4 patients (9.5%), dopaminergic drug use in 4 patients (9.5%), and use of typical antipsychotics alone in 2 patients (4.8%). In 3 patients (7.1%), no causative agent could be identified. Evaluation of medical history revealed psychotic disorders in 17 patients (40.0%), mood disorders in 12 patients (29.0%), other psychiatric disorders in 7 patients (17.0%), and neurodegenerative disorders in 6 patients (14.0%) (Table 1).

On clinical assessment, hyperthermia (body temperature >38 °C) was observed in 20 patients (47.6%). Tachycardia was the most frequently observed autonomic finding, present in 26 patients (61.9%). Hypotension was observed in 4 patients (9.5%), while hypertension was observed in 5 patients (11.9%). Altered mental status (GCS <15) was present in the majority of cases and was detected in 39 patients (92.9%). Muscle rigidity was observed in 11 patients (26.2%).

Laboratory analysis revealed creatine kinase levels greater than 1000 U/L in 24 patients (57.1%) and leukocytosis (WBC > 10,000/mm^3^) in 30 patients (71.4%). Among severity-of-illness scores, the median APACHE II score was 16.75 (12.00–20.75), the median SOFA score was 6.00 (3.00–9.00), and the median Glasgow Coma Scale score was 8.50 (5.00–13.50). Detailed data on clinical and laboratory findings, as well as descriptive statistics for all continuous variables, are presented in Table 2.

The median length of stay in the intensive care unit was 6.77 days (IQR: 2.96–10.28). During the ICU stay, 25 patients (59.5%) required mechanical ventilation. Acute kidney injury developed in 13 patients (31.0%), and continuous renal replacement therapy (CRRT) was administered to 9 patients (21.4%). ICU mortality was 9.5%, with 4 deaths observed in the study population. Among the 9 patients who received CRRT, 3 patients (33.3%) died in the ICU. In the treatment of NMS, bromocriptine was used in 23 patients (54.8%), dantrolene in 2 patients (4.8%), and amantadine in 5 patients (11.9%). Data regarding intensive care management and outcome measures are summarized in Table 3.

Data are presented as median (interquartile range) for continuous variables and as n (%) for categorical variables.

Continuous variables are presented as median (interquartile range) or mean ± standard deviation, as originally reported. Categorical variables are presented as n (%).

Continuous variables are presented as median (interquartile range). Categorical variables are presented as n (%).

## 4. Discussion

In this retrospective cohort study, the clinical characteristics, intensive care management, mortality, and clinical outcomes of 42 patients with NMS treated in an adult intensive care unit were evaluated. The main findings of our study were as follows: (i) an ICU mortality rate of 9.5%, (ii) a 21% requirement for CRRT with a mortality rate of 33% among patients requiring CRRT, (iii) a mechanical ventilation requirement of approximately 60%, with variable morbidity and mortality, and (iv) the absence of concurrent classical NMS findings in all cases, particularly the lower observed rates of hyperthermia and muscle rigidity.

The pathophysiology of NMS is still not clearly understood. There are several theories involving central dopamine receptor blockade in the hypothalamus, which may lead to hyperthermia and dysautonomia, as well as interference with nigrostriatal dopamine pathways, which may result in rigidity and tremor [21,22]. Other neurotransmitter systems, including epinephrine, serotonin, and acetylcholine, are also involved [23]. Alternative theories have been proposed regarding impaired modulation of the sympathetic nervous system, direct alterations in muscle mitochondrial function, and genetic susceptibility to the disease [24,25]. However, these theories fail to explain all clinical manifestations and render NMS a disorder of unknown etiology.

Mortality associated with NMS was previously reported to exceed 30%, but in recent years, with increased clinician awareness and the introduction of newer antipsychotic agents, this rate has decreased to approximately 5–10% [26]. The mortality rate associated with NMS in Türkiye has been reported as 13.9% [27]. In our study population, ICU mortality was found to be 9.5%. This decrease may be attributed to early diagnosis and advances in intensive care supportive treatments. Moreover, considering that our study population consisted of critically ill patients treated in the intensive care unit, a higher mortality rate compared with psychiatric wards would be an expected finding. In our study, 61.9% of patients diagnosed with NMS were male, which is very similar to other reported case series [28]. The higher frequency of NMS in males may be related to the greater prevalence of psychotic disorders in men and, consequently, more frequent use of antipsychotic medications [29].

In the consensus, scores were assigned to the diagnostic criteria for NMS; however, no cutoff value was defined for diagnosis. According to the DSM-5, Neuroleptic Malignant Syndrome is an acute-onset and potentially fatal syndrome characterized by hyperthermia, generalized muscle rigidity, altered mental status, and autonomic instability, typically occurring after the initiation or dose escalation of dopamine D_2_ receptor antagonist antipsychotics or following the abrupt discontinuation of dopaminergic medications [27,30]. We did not identify any studies regarding the clinical implementation of the criteria defined in the consensus. Therefore, we determined the diagnoses of our patients according to the DSM-5. Because the clinical course, symptoms, and laboratory tests are highly heterogeneous, the diagnosis of this syndrome is difficult [21]. Serotonin syndrome, malignant hyperthermia, and infection, which are included in the differential diagnosis of NMS, must be excluded. The diagnosis of NMS is still a diagnosis of exclusion. Differential diagnosis of hyperthermia and altered mental status is required, because hyperthermia and altered mental status due to infection may be confused with NMS [31]. The differential diagnosis of NMS includes serotonin syndrome, malignant hyperthermia, and malignant catatonia. Serotonin syndrome is distinguished from NMS by a history of serotonin reuptake inhibitor use, hyperreflexia, and myoclonus. Malignant hyperthermia requires exposure to halogenated inhalational anesthetics and/or the use of the depolarizing neuromuscular blocking agent succinylcholine. Distinctive features of malignant catatonia, compared with NMS, include increased positive symptoms and a behavioral prodrome such as automatism, agitation, or psychosis [30].

In our patients, hyperthermia was observed in 47.6%, rigidity in 26.2%, autonomic instability in 69% (defined as the presence of hypotension, hypertension, or tachycardia), and altered mental status in 92.9%. Although studies in the literature have reported the frequency of rigidity to range between 91% and 96%, there are also reports in which rigidity was not detected in some cases [14,32]. In this study, rigidity was observed in 26.2% of patients. The lower rate of rigidity compared with those reported in the literature may be related to early sedation and analgesia, mechanical ventilation, and aggressive supportive treatments applied in the intensive care setting, which may mask motor findings. In addition, since one of the indications for ICU admission in these cases was a low Glasgow Coma Scale score, the high prevalence of altered mental status represents a clinically expected finding.

Among laboratory findings, CK levels and leukocytosis support the diagnosis. Elevated CK levels are one of the most reliable findings for evaluating the development of NMS. In a study conducted in a psychiatric ward, elevated CK levels were present in 92% of patients [33]. In this study, elevated CK levels were present in 57% of patients. This may be interpreted as the result of intravenous hydration initiated before ICU admission and early supportive therapy, which may have reduced the severity of rhabdomyolysis. However, since normal or mildly elevated CK levels do not exclude NMS, diagnosis should not be reduced to laboratory thresholds alone in the presence of clinical suspicion. A case–control study demonstrated that patients with NMS were more likely to have elevated CK levels during prior non-NMS hospitalizations compared with controls (76% vs 30%) [34]. Another laboratory finding is leukocytosis, with the white blood cell count typically ranging between 10,000 and 40,000/mm^3^, and a left shift may be present [4,14,15]. In our study, leukocytosis was present in 71% of patients. This finding supports the existing literature.

The main approach in the management of patients with NMS includes discontinuation of the offending agent, intensive supportive care, and pharmacological and/or electroconvulsive therapies in selected cases. Intensive supportive care may include hemodynamic stabilization, aggressive hydration, and temperature control, as well as advanced organ support ranging from invasive mechanical ventilation in cases of clinical deterioration to renal replacement therapy (CRRT/hemodialysis) in patients who develop rhabdomyolysis and acute kidney injury [35]. In a study including 405 case reports, the effect of specific NMS pharmacotherapy (dantrolene, bromocriptine) and ECT on mortality was investigated. While no significant difference was observed in mild and moderate cases, mortality was significantly lower in severe NMS compared with symptomatic treatment alone (*p* = 0.018) [28]. In this study, bromocriptine was administered to 54.8% of patients with NMS treated in the intensive care unit, and dantrolene was administered to 4.8%, while no patient received ECT. Because our study was descriptive in nature and had a limited sample size, it was not possible to comparatively evaluate the effect of specific pharmacotherapies on mortality. However, the observed low ICU mortality rate (9.5%) suggests a clinical course consistent with this case series.

A notable finding regarding intensive care management in our study was that 60% of patients (n = 25) required mechanical ventilation. In addition, acute kidney injury developed in 31% of patients (n = 13), and 21% (n = 9) required CRRT. Mortality among patients who received CRRT was 33% (3/9). This finding suggests that the subgroup with renal dysfunction may have had a more severe clinical phenotype. Considering that rhabdomyolysis, hemodynamic instability, and intense metabolic stress may contribute to acute kidney injury in NMS, the need for renal replacement therapy may represent a marker of disease severity and prognosis. Further studies with larger patient populations are needed in this area.

In a study focusing specifically on intensive care management in patients with NMS, mechanical ventilation was applied in 45% of patients. Acute kidney injury was observed in 40% of cases, and rhabdomyolysis was reported in 75% of patients. None of the patients required CRRT [3]. In a nationwide hospital discharge sample conducted in the United States between 2002 and 2011, a total of 1346 cases were analyzed, and the most common complication was rhabdomyolysis (30.1%). Other frequent complications included acute respiratory failure (16.1%), acute kidney failure (17.7%), sepsis (6.2%), and other systemic infections [36]. Melli et al. reported that acute renal failure developed in 34% of patients with NMS-associated rhabdomyolysis [37]. In Japan, Sanai et al. reported six patients who developed myoglobinuria and acute kidney injury secondary to rhabdomyolysis and demonstrated that these patients were successfully treated with hemodialysis and dantrolene [38]. The low rate of dantrolene use in our study (4.8%) may be related not to the effectiveness of the treatment but rather to our center’s clinical practice patterns, drug availability, and the preferences of intensive care physicians.

In our study, 69% of patients were using atypical antipsychotics, 4.8% typical antipsychotics, 9.5% combined typical and atypical antipsychotics, and 9.5% dopaminergic medications. This finding indicates that NMS is not a condition exclusive to typical antipsychotics. The presence of cases associated with dopaminergic drug exposure or withdrawal also demonstrates that the spectrum of NMS is not limited to psychiatric patient populations, and that neurodegenerative diseases and changes in dopaminergic therapy may represent important triggers. In our study, six patients developed NMS following discontinuation or modification of dopaminergic medications while being hospitalized with a diagnosis of neurodegenerative disease. However, only four of these patients were receiving active dopaminergic therapy at the time of ICU admission, as dopaminergic treatment had already been discontinued or modified prior to ICU transfer in the remaining cases. Although this subgroup was small in absolute number, it constituted 14% of the study population. In a study conducted by Takubo et al. involving 93 patients with Parkinson’s disease diagnosed with NMS, the most common triggering factor was discontinuation or dose reduction in dopaminergic therapy. During dopaminergic treatment, an increase in body temperature should be considered NMS until proven otherwise [39]. There are studies suggesting that this condition developing in patients with Parkinson’s disease should be referred to as malignant syndrome rather than neuroleptic malignant syndrome and that it should be evaluated as a distinct entity [40].

Neuroleptic Malignant Syndrome is a rare emergency associated with a high risk of mortality; therefore, no randomized controlled trials were identified in the literature. Available evidence is largely based on case reports, case series, and review articles. For this reason, to the best of our knowledge, our study represents the only study including 42 patients and focusing exclusively on patients managed in the intensive care unit.

Finally, the single-center and retrospective design of our study, as well as the inclusion of only intensive care patients, may limit its generalizability. Data regarding treatments administered for NMS prior to ICU admission were not available. Nevertheless, the exclusive focus on the ICU population, the inclusion of detailed clinical and laboratory variables, and the concurrent reporting of advanced organ support requirements such as CRRT and mechanical ventilation may be considered strengths of the study.

## 5. Conclusions

In this retrospective single-center study, the clinical outcomes, intensive care management, and mortality of 42 patients with NMS treated in an adult intensive care unit were evaluated. Our findings demonstrate that NMS is a heterogeneous syndrome in intensive care practice, characterized by a high requirement for organ support and the absence of concurrent classical findings in all cases. ICU mortality in our cohort was 9.5%, with invasive mechanical ventilation required in 60% of patients and CRRT in 21%. Notably, the higher mortality observed among patients requiring CRRT (3/9; 33%) suggests that renal dysfunction may represent an important marker of clinical severity in NMS.

In conclusion, in the presence of suspected NMS, rapid discontinuation of the offending agent, prompt initiation of intensive supportive care, and early recognition of potential complications such as respiratory failure, rhabdomyolysis, and acute kidney injury are of critical importance. Given that NMS may occur in the absence of hyperthermia or prominent rigidity, the threshold for clinical suspicion should remain low, and diagnosis and management should be conducted using a multidisciplinary approach. Multicenter studies with larger sample sizes are needed to validate these findings and to better define prognostic markers in NMS.

## Figures and Tables

**Table 1:** Baseline Characteristics of ICU Patients with Neuroleptic Malignant Syndrome.

Variable	Value
Demographics	
Number of patients, n	42
Age, years	48.0 (24.1–75.5)
Male sex, n (%)	26 (61.9%)
Causative agent/Medication exposure	
Atypical antipsychotic	29 (69.0%)
Typical + atypical antipsychotic	4 (9.5%)
Dopaminergic drugs	4 (9.5%)
Typical antipsychotic alone	2 (4.8%)
Unknown	3 (7.1%)
Medical history	
Psychotic disorders	17 (40.5%)
Mood disorders	12 (28.6%)
Other psychiatric disorders	7 (16.7%)
Neurodegenerative disorders	6 (14.3%)

**Table 2:** Clinical and Laboratory Findings at ICU Admission.

Variable	Value
Clinical findings	n (%)
Hyperthermia (temperature > 38 °C)	20 (47.6%)
Tachycardia (≥100 beats/min)	26 (61.9%)
Hypotension (MAP ≤ 65 mmHg)	4 (9.5%)
Hypertension (MAP ≥ 110 mmHg)	5 (11.9%)
Altered mental status (GCS < 15)	39 (92.9%)
Muscle rigidity	11 (26.2%)
Creatine kinase >1000 U/L	24 (57.1%)
Leukocytosis (WBC > 10.0 × 10^9^/L)	30 (71.4%)
Laboratory and physiological variables	Median (IQR)
Blood urea nitrogen	48 (24.10–75.50)
Creatinine	0.90 (0.69–1.43)
Glomerular filtration rate	64.75 (11.88–115.25)
White blood cell count, ×10^9^/L	11.96 (9.49–14.47)
Creatine kinase, U/L	1957.50 (129.50–4130.25)
Severity scores	Mean (min–max)
APACHE II score	16.8 (2–38)
SOFA score	6.0 (1–18)
Glasgow Coma Scale	8.5 (3–15)
Vital signs at admission	Mean ± SD
Body temperature, °C	37.80 (37.10–38.77)
Heart rate, beats/min	108.75 ± 23.31
Oxygen saturation, %	94.42 (92.58–96.56)
Systolic arterial pressure, mmHg	120.51 ± 24.45
Diastolic arterial pressure, mmHg	65.40 ± 15.07
Mean arterial pressure, mmHg	83.64 ± 17.21

**Table 3:** Intensive Care Management and Outcomes.

Variable	Value
Mechanical ventilation	25 (59.5%)
Acute kidney injury	13 (31.0%)
Continuous renal replacement therapy (CRRT)	9 (21.4%)
Bromocriptine use	23 (54.8%)
Dantrolene use	2 (4.8%)
Amantadine use	5 (11.9%)
ICU mortality	4 (9.5%)
	Median (IQR)
Length of ICU stay, days	6.77 (2.96–10.28)
Duration of mechanical ventilation, days	0.85 (0.00–4.21)

## Data Availability

The data presented in this study are available from the corresponding author upon reasonable request. The data are not publicly available due to ethical restrictions.

## Supplementary Materials

The following supporting information can be downloaded at: https://www.mdpi.com/article/10.3390/medicina62020378/s1, Supplementary Table S1. Individual demographic, clinical, laboratory, and outcome characteristics of adult patients with neuroleptic malignant syndrome admitted to the intensive care unit.

## Abbreviations

NMS	Neuroleptic Malignant Syndrome
ICU	Intensive Care Unit
MV	Mechanical Ventilation
AKI	Acute Kidney Injury
CRRT	Continue Renal Replacement Therapy

## References

[1] Nakamura M., Yasunaga H., Miyata H., Shimada T., Horiguchi H., Matsuda S. (2012). Mortality of neuroleptic malignant syndrome induced by typical and atypical antipsychotic drugs: A propensity-matched analysis from the Japanese Diagnosis Procedure Combination database. J. Clin. Psychiatry.

[2] Keck P.E., Pope H.G., Cohen B.M., McElroy S.L., Nierenberg A.A. (1989). Risk factors for neuroleptic malignant syndrome: A case-control study. Arch. Gen. Psychiatry.

[3] Lopez O., Rabinstein A.A., Wijdicks E.F.M. (2025). Contemporary perspectives in critical care of neuroleptic malignant syndrome. Neurocrit. Care.

[4] Caroff S.N., Mann S.C. (1993). Neuroleptic malignant syndrome. Med. Clin. N. Am..

[5] Seitz D.P., Gill S.S. (2009). Neuroleptic malignant syndrome complicating antipsychotic treatment of delirium or agitation in medical and surgical patients: Case reports and a review of the literature. Psychosomatics.

[6] Kogoj A., Velikonja I. (2003). Olanzapine-induced neuroleptic malignant syndrome: A case review. Hum. Psychopharmacol..

[7] Schultz A.R., Singh S., Linek-Rajapaksha C.E., Goode H.R., Fusick A.J. (2024). Neuroleptic malignant syndrome in the context of lithium toxicity and aripiprazole use. Clin. Neuropharmacol..

[8] Tee Z.J. (2024). Prochlorperazine-induced neuroleptic malignant syndrome. Am. J. Emerg. Med..

[9] Manabe S., Yanagi H., Ozawa H., Takagi A. (2019). Neuroleptic malignant syndrome as part of an akinetic crisis associated with sepsis in a patient with Lewy body disease. BMJ Case Rep..

[10] Jamshidi N., Dawson A. (2019). The hot patient: Acute drug-induced hyperthermia. Aust. Prescr..

[11] Gurrera R.J., Caroff S.N., Cohen A., Carroll B.T., DeRoos F., Francis A., Frucht S., Gupta S., Levenson J.L., Mahmood A. (2011). An international consensus study of neuroleptic malignant syndrome diagnostic criteria using the Delphi method. J. Clin. Psychiatry.

[12] American Psychiatric Association (2022). Diagnostic and Statistical Manual of Mental Disorders.

[13] Velamoor V.R., Norman R.M.G., Caroff S.N., Mann S.C., Sullivan K.A., Antelo R.E. (1994). Progression of symptoms in neuroleptic malignant syndrome. J. Nerv. Ment. Dis..

[14] Levenson J.L. (1985). Neuroleptic malignant syndrome. Am. J. Psychiatry.

[15] Rosebush P., Stewart T. (1989). A prospective analysis of 24 episodes of neuroleptic malignant syndrome. Am. J. Psychiatry.

[16] Wilkinson R., Meythaler J.M., Guin-Renfroe S. (1999). Neuroleptic malignant syndrome induced by haloperidol following traumatic brain injury. Brain Inj..

[17] Szota A.M., Radajewska I., Grudzka P., Araszkiewicz A. (2020). Lamotrigine-, quetiapine- and aripiprazole-induced neuroleptic malignant syndrome in a patient with lithium-related renal failure. BMC Psychiatry.

[18] Caroff S.N., Roberts C.B., Rosenberg H., Tobin J.R., Watt S., Mashman D., Riazi S., Berkowitz R.M. (2022). Intravenous dantrolene in hypermetabolic syndromes: A survey of the U.S. Veterans Health Administration database. BMC Anesthesiol..

[19] Trollor J.N., Sachdev P.S. (1999). Electroconvulsive treatment of neuroleptic malignant syndrome: A review and report of cases. Aust. N. Z. J. Psychiatry.

[20] Wang H.C., Hsieh Y. (2001). Treatment of neuroleptic malignant syndrome with subcutaneous apomorphine monotherapy. Mov. Disord..

[21] Maia A., Cotovio G., Barahona-Corrêa B., Oliveira-Maia A.J. (2021). Diagnosis and treatment of neuroleptic malignant syndrome in the intensive care unit. Acta Med. Port..

[22] Boulant J.A. (2000). Role of the preoptic-anterior hypothalamus in thermoregulation and fever. Clin Infect Dis..

[23] Spivak B., Maline D.I., Vered Y., Kozyrev V.N., Mester R., Neduva S.A., Ravilov R.S., Graff E., Weizman A. (2000). Circulatory catecholamines and serotonin in neuroleptic malignant syndrome. Acta Psychiatr. Scand..

[24] Mihara K., Kondo T., Suzuki A., Yasui-Furukori N., Ono S., Sano A., Koshiro K., Otani K., Kaneko S. (2003). Dopamine D2 and D3 receptor gene polymorphisms and neuroleptic malignant syndrome. Am. J. Med. Genet. B.

[25] Gurrera R.J. (2002). Is neuroleptic malignant syndrome a neurogenic form of malignant hyperthermia?. Clin. Neuropharmacol..

[26] UpToDate Neuroleptic Malignant Syndrome. https://www.uptodate.com/contents/neuroleptic-malignant-syndrome.

[27] Velamoor V.R. (1998). Neuroleptic malignant syndrome: Recognition, prevention and management. Drug Saf..

[28] Kuhlwilm L., Schönfeldt-Lecuona C., Gahr M., Connemann B.J., Keller F., Sartorius A. (2020). Neuroleptic malignant syndrome: A systematic case series analysis. Acta Psychiatr. Scand..

[29] Caton C.L.M., Xie H., Drake R.E., McHugo G. (2014). Gender differences in psychotic disorders with concurrent substance use. J. Dual Diagn..

[30] Wijdicks E.F.M., Ropper A.H. (2024). Neuroleptic malignant syndrome. N. Engl. J. Med..

[31] Costa M., Covas A.R., Correia F.N., Bernardo S., Silveira P. (2025). Neuroleptic malignant syndrome requiring intensive care unit admission in patients with SARS-CoV-2 infection. Acute Crit. Care.

[32] Strawn J.R., Keck P.E., Caroff S.N. (2007). Neuroleptic malignant syndrome. Am. J. Psychiatry.

[33] Oneib B., Zaimi O. (2021). Neuroleptic malignant syndrome: Clinical expression, complications, and atypical presentation. Middle East. Curr. Psychiatry.

[34] Hermesh H., Manor I., Shiloh R., Aizenberg D., Benjamini Y., Munitz H., Weizman A. (2002). High serum creatine kinase as a possible risk factor for neuroleptic malignant syndrome. J. Clin. Psychopharmacol..

[35] Macedo E., Rodrigues F., Marques A.R., Ribeiro L., Silveira P. (2024). Neuroleptic malignant syndrome with exceptionally high creatine kinase and myoglobin levels. Cureus.

[36] Modi S., Dharaiya D., Schultz L., Varelas P. (2016). Neuroleptic malignant syndrome: Complications, outcomes, and mortality. Neurocrit. Care.

[37] Melli G., Chaudhry V., Cornblath D.R. (2005). Rhabdomyolysis: An evaluation of 475 hospitalized patients. Medicine.

[38] Sanai T., Matsui R., Hirano T., Torichigai S., Yotsueda H., Higashi H., Hirakata H., Iida M. (2006). Successful treatment of six patients with Neuroleptic malignant syndrome associated with myoglobulinemic acute renal failure. Ren. Fail..

[39] Takubo H., Harada T., Hashimoto T., Inaba Y., Kanazawa I., Kuno S., Mizuno Y., Mizuta E., Murata M., Nagatsu T. (2003). A collaborative study on the Malignant syndrome in Parkinson’s disease and related disorders. Park. Relat. Disord..

[40] Mizuno Y., Takubo H., Mizuta E., Kuno S. (2003). Malignant syndrome in Parkinson’s disease: Concept and review of the literature. Park. Relat. Disord..

