# Supplementary material for: Intensive Care Management and Outcomes of Neuroleptic Malignant Syndrome: A Single-Center Retrospective Study
Source: Medicina (Kaunas). 2026 Feb 14;62(2):378. doi: 10.3390/medicina62020378 (PMC12942336; doi:10.3390/medicina62020378)
Supplement: Supplementary file 1 [file medicina-62-00378-s001.zip › medicina-4146179-supplementary.pdf]

Supplementary Table S1. Individual demographic, clinical, laboratory, and outcome characteristics of adult patients with neuroleptic malignant syndrome admitted to the intensive care unit.

Patient	Sex/Age	Neuroleptic agent(s)	Notable laboratory test results	AKI	CRRT	MV	Drug treatment	Outcome
1	F,21	None	WBC: 9.79, CPK: 1238	Yes	Yes	Yes	LORAZEPAM	Death
2	M,38	None	WBC: 7.94, CPK: 87	No	No	Yes	LORAZEPAM	Recovered
3	F,48	None	WBC: 9.39, CPK: 93	No	No	No	None	Recovered
4	F,32	haloperidol,risperidon	WBC: 13.07, CPK: 4893	No	No	No	LORAZEPAM, BROMOKRIPTIN	Recovered
5	M,37	haloperdol, ketiapin	WBC: 10.6, CPK: 2535	No	No	No	None	Recovered
6	M,79	ketiapin, haloperidol	WBC: 7.49, CPK: 237	No	No	No	None	Recovered
7	M,18	ketiapin olanzapin risperidon	WBC: 10.02, CPK: 3169	No	No	No	BROMOKRIPTIN	Recovered
8	M,35	ketiapin, haloperidol	WBC: 6.84, CPK: 131	No	No	No	None	Recovered
9	M,63	haloperdol	WBC: 8.15, CPK: 775	No	No	Yes	LORAZEPAM, BROMOKRIPTIN	Recovered
10	F,68	lityum,haloperidol	WBC: 15.56, CPK: 2649	Yes	Yes	Yes	BROMOKRIPTIN	Death
11	M,50	risperidon,olanzapin	WBC: 13.86, CPK: 8149	Yes	Yes	Yes	BROMOKRIPTIN	Recovered
12	M,30	ketiapin olanzapin risperidon	WBC: 14.21, CPK: 3045	Yes	Yes	Yes	None	Recovered

13	M,49	ketiapin	WBC: 11.41, CPK: 91	Yes	Yes	No	None	Recovered
14	M,43	risperidon	WBC: 46.82, CPK: 101	Yes	Yes	Yes		Recovered
15	M,63	risperidon, ketiapin	WBC: 11.53, CPK: 744	Yes	Yes	No	AMANTADIN,DIAZEPAM,BROMOKRIPTIN	Recovered
16	F,31	risperidon	WBC: 14.52, CPK: 62	No	No	Yes	None	Recovered
17	F,45	ketiapin, risperidon	WBC: 10.84, CPK: 117	No	No	No	None	Recovered
18	F,50	olanzapin,paliperidon	WBC: 14.31, CPK: 59	No	No	Yes	None	Recovered
19	F,22	olanzapin	WBC: 28.08, CPK: 6046	No	No	Yes	LORAZEPAM,BROMOKRIPTIN	Recovered
20	M,35	paliperidon	WBC: 11.92, CPK: 3006	No	No	Yes	LORAZEPAM,BROMOKRIPTIN	Recovered
21	M,57	risperidon	WBC: 15.99, CPK: 3765	No	No	Yes	BROMOKRIPTIN	Recovered
22	F,23	risperidon	WBC: 7.79, CPK: 67	No	No	No	None	Recovered
23	M,39	ketiapin	WBC: 20.23, CPK: 141	Yes	No	Yes	None	Death
24	M,32	None	WBC: 6.31, CPK: 10215	No	No	Yes	BROMOKRIPTIN	Recovered
25	F,75	ketiapin	WBC: 10.99, CPK: 129	No	No	Yes	None	Recovered
26	F,42	risperidon	WBC: 8.92, CPK: 52	No	No	No	None	Recovered
27	F,31	ketiapin, paliperidon	WBC: 6.71, CPK: 1709	No	No	No	None	Recovered

28	M,51	klozapin, trifluperazin,paliperidon	WBC: 13.61, CPK: 6927	No	No	No	BROMOKRIPTIN	Recovered
29	M,67	risperidon, ketiapin	WBC: 13.79, CPK: 6052	No	No	Yes	BROMOKRIPTIN	Recovered
30	M,84	olanzapin	WBC: 12.4, CPK: 2301	No	No	No	AMANTADIN, LEVODOPA, BROMOKRIPTIN	Recovered
31	M,73	ketiapin , paliperidon	WBC: 13.64, CPK: 4359	Yes	No	Yes	AMANTADIN, BROMOKRIPTIN	Recovered
32	M,50	olanzapin	WBC: 6.57, CPK: 1011	No	No	Yes	BROMOKRIPTIN	Recovered
33	M,68	ketiapin	WBC: 12, CPK: 81	No	No	Yes	KARBAMEZEPİN, BROMOKRIPTIN	Recovered
34	F,74	risperidon	WBC: 16.99, CPK: 7073	No	No	Yes	LORAZEPAM, DANTROLEN, BROMOKRIPTIN	Recovered
35	M,26	ketiapin	WBC: 7.75, CPK: 4240	No	No	Yes	DİAZEPAM, BROMOKRIPTIN	Recovered
36	F,64	ketiapin, biperiden hidroklorür	WBC: 17, CPK: 666	Yes	No	No	DİAZEPAM, BROMOKRIPTIN	Recovered
37	M,40	ketiapin	WBC: 33.2, CPK: 936	Yes	No	Yes	BROMOKRIPTIN	Recovered
38	M,52	paliperidon	WBC: 16.76, CPK: 2206	No	No	Yes	BROMOKRIPTIN	Recovered
39	F,37	levodopa, amantadin	WBC: 11.73, CPK: 34624	Yes	Yes	Yes	DANTROLEN	Recovered
40	M,53	ropinirol,levodopa,resajilin, amantadin	WBC: 13.52, CPK: 3801	Yes	Yes	Yes	BROMOKRIPTIN	Death
41	M,72	levodopa, amantadin	WBC: 10.3, CPK: 2537	No	No	No	AMANTADIN, LEVODOPA, BROMOKRIPTIN	Recovered
42	F,77	amantadin, rasajilin, ketiapin	WBC: 16.43, CPK: 4572	No	No	No	AMANTADIN, RESAJİLİN,	Recovered
